# Immunophenotyping of Sheep Paraffin-Embedded Peripheral Lymph Nodes

**DOI:** 10.3389/fimmu.2018.02892

**Published:** 2018-12-11

**Authors:** Eleonora Melzi, Mara S. Rocchi, Gary Entrican, Marco Caporale, Massimo Palmarini

**Affiliations:** ^1^MRC-University of Glasgow Centre for Virus Research, Glasgow, United Kingdom; ^2^Moredun Research Institute, Penicuik, United Kingdom; ^3^Istituto Zooprofilattico Sperimentale dell'Abruzzo e del Molise “G. Caporale”, Teramo, Italy

**Keywords:** sheep, lymph node, antibody, immunohistochemistry, confocal microscopy, dendritic cell, stromal cell, macrophages

## Abstract

Sheep are not only a major livestock species globally, they are also an important large animal model for biomedical research and have contributed to our understanding of the ontogeny and architecture of the mammalian immune system. In this study, we applied immunohistochemistry and multicolor immunofluorescence in fixed and paraffin-embedded lymph nodes to phenotype the key populations of antigen presenting cells, lymphocytes, and stromal cells that orchestrate the host adaptive immune response. We used an extensive panel of antibodies directed against markers associated with dendritic cells (MHC class II, CD83, and CD208), macrophages (CD11b, CD163, and CD169), stromal cells (CNA.42, S100, and CD83), and lymphocytes (CD3, Pax5, CD4, CD8). Using different methods of tissue fixation and antigen retrieval, we provide a detailed immunophenotyping of sheep lymph nodes including the identification of potential subpopulations of antigen presenting cells and stromal cells. By characterizing cells expressing combinations of these markers in the context of their morphology and location within the lymph node architecture, we provide valuable new tools to investigate the structure, activation, and regulation of the sheep immune system in health and disease.

## Introduction

For more than 10,000 years domestic ruminants have played an important role in most human societies as a source of meat, milk, and dairy products, textile, and labor. In developing countries, cattle, sheep, and goats remain one of the main sources of livelihoods for millions of poor smallholder farmers (1, 2). In more industrialized countries, the livestock sector is increasingly organized in long marketing supply chains that employ at least 1.3 billion people globally and accounts for 53% of agricultural GDP (2). For these reasons, diseases affecting animal health can have a profound impact on the global economy. The majority of the currently emerging human pathogens are of animal origin. Small ruminants can transmit a variety of diseases to humans such as brucellosis, leptospirosis, listeriosis, Q Fever, chlamydophilosis, and tuberculosis (3) and act as reservoirs for emerging arboviral infections that impact on human health such as Rift Valley Fever (mosquito-borne) and Crimean Congo haemorrhagic fever (tick borne). These diseases all involve complex host-pathogen interactions that need to be dissected for the development of safe and effective control strategies, including novel vaccines.

Due to its size and accessibility, the sheep has been used as a suitable biomedical model to understand fundamental immunological mechanisms. For example, *in utero* thymectomy in fetal lamb revealed the ontogeny of T cell development (4) while lymphatic cannulation in adult sheep has been essential to our understanding of lymphoid and myeloid cell recirculation and compartmentalization (5). These studies advanced our capability to conduct basic ovine immunology, most notably through the production of cell-subset specific monoclonal antibodies (6). While there have been many improvements, the tools to study the sheep immune responses remain relatively limited in relation to those available for mice (7–12).

Therefore, while mice remain the biomedical model of choice for studying a variety of human and animal diseases, it is unrealistic to expect genetically manipulated “custom-made” mouse strains to be representative of every aspect of the intricate interplay between a pathogen and his host. Pathogen-host interactions are influenced by their co-evolutionary history. Hence, observations made in mouse models of disease do not necessarily recapitulate the interactions between pathogens and their natural host (13, 14). Large animals like sheep can provide a unique opportunity to study naturally occurring diseases in their target species both in the field and in experimental conditions; hence the community need for improved immunological tools.

Imaging techniques such as immunohistochemistry and immunofluorescence allows the identification of cellular markers in the context of their anatomical location. These techniques provide unique information on cellular interactions within the architecture of the tissues and are synergistic to flow cytometry which is instead a more robust method to provide quantitative data on a large number of cells. As part of a previously published study (15), we described sheep lymph nodes (LNs) infected by bluetongue virus to define the cellular changes that adversely affect the development of host immune responses. LNs are crucial lymphoid organs for antigen presentation, and for the subsequent development of an adaptive immune response able to counteract infections. Therefore, we have evaluated more than fifty monoclonal and polyclonal antibodies, in order to identify markers able to recognize distinct cell types in fixed and paraffin-embedded sheep LNs. Our study will facilitate further research needing to define the anatomy and compartmentalization of the ovine peripheral LNs in basic and applied immunological studies in sheep.

## Materials and Methods

### Animals

Sheep LNs were sourced at the Istituto Zooprofilattico Sperimentale dell'Abruzzo e del Molise “G. Caporale” (Teramo, Italy) in accordance with locally and nationally approved protocols regulating animal experimental use (protocol numbers 7440; 11427; 12301). Skin-draining LNs (prescapular, retromandibular, inguinal, and popliteal) were collected from 10 healthy sheep (Sardinian or mixed breed) during post-mortem examination.

### Preparation of Tissues

Tissue samples were cut sagittally and placed into processing cassettes. The cassettes were immersed in either a 10% neutral buffered formalin solution (Sigma, United Kingdom) or a 1% zinc salts fixative solution (at a ration 10: 1 solution volume/sample volume; BD Pharmingen) and allowed to sit for 24–48 h at room temperature before processing. After 48 h, tissues were removed from the fixative solutions, dehydrated in increasing concentration of ethanol (from 0 to 100%), cleared in xylene and embedded in paraffin blocks as per standard histology protocols.

### Preparation of Sections for Labeling

Tissue sections were cut with a microtome (4 μm thickness) and mounted on microscope slides. Sections were deparaffinised with multiple passages in xylene, re-hydrated in decreasing concentration of ethanol and then rinsed in water.

### Antigen Retrieval

Different types of antigen retrieval techniques were tested on formalin-fixed, paraffin embedded tissues to unmask specific epitopes. For heat-induced epitope retrieval (HIER), sections were treated with Access Retrieval Unit (Menarini) in sodium citrate buffer (pH 6), for 1 min 30 s at 125°C at full pressure, then rinsed in Tris buffer pH 7.5. For protease induced epitope retrieval (PIER), proteolytic digestion of formalin-fixed tissues was performed using either a ready-to-use solution of proteinase-K in 0.05 M Tris-HCl, 0.015 M sodium azide, pH 7.5 (Dako, United Kingdom) for 10 min at 37°C, or a trypsin solution (0.05% trypsin, 1% CaCl2, and 0.05% chymotrypsin) pH 7.8 (Sigma, United Kingdom) for 20 min at 37°C and then rinsed with PBS.

### Immunohistochemistry (IHC)

After antigen retrieval, the tissue sections were permeabilised with a PBS solution of 1% Triton-X (Sigma) for 10 min at room temperature (RT). Slides were then treated for 30 min at RT with 3% hydrogen peroxide in PBS to quench endogenous peroxidase activity. After washing 3 times with PBS 0.05% Tween 20 (PBS-T20), the sections were incubated for 80 min with a blocking buffer containing 5% bovine serum albumin in PBS to block unspecific binding sites. Primary antibodies specific or cross-reactive to sheep antigens (Table 1) were diluted in blocking buffer and incubated with the tissue sections overnight at 4°C. Excess of primary antibodies was removed by washing with PBS-T20. For primary antibody detection, sections were incubated with appropriate isotype specific secondary antibodies conjugated with horseradish peroxidase (HRP) for 1 h at RT. This was followed by a 5 min incubation with 3,3′-diaminobenzidine (DAB) substrate-chromogen (EnVision+ System, Dako). Tissues were counterstained using Mayer's haematoxylin and mounted with clear resin and coverslips for long-term storage. Tissue sections were screened using a bright field microscope (Olympus) and images captured using cell^∧^D software (Olympus).

**Table 1:** List of antibodies successfully used in this study.

**Specificity**	**Ab Clone**	**Host/Target^**a**^**	**Isotype^**b**^**	**Source^**c**^**	**Results^**d**^**	**Known cross reactivity in sheep (References)^‡^**	**AA identity with sheep orthologue (%)**	**Original References**
CD3^†^	F7.2.38	Mo α Hu	IgG_1_	Dako	F	Tested in ruminants (16, 17)	92^*^	(18)
WC1	CC15	Mo α Bv	IgG_2a_	Bio-Rad	F	Yes (8)	–	(19)
CD8	CC63	Mo α Bv	IgG_2a_	Bio-Rad	Z	Yes (8)	–	(20)
CD8	38.65	Mo α Sh	IgG_2a_	Bio-Rad	Z	–	–	(21)
CD4	44.38	Mo α Sh	IgG_2a_	Bio-Rad	Z	–	–	(21)
CD21 (CR2)	CC21	Mo α Bv	IgG_1_	Bio-Rad	Z	Yes (22)	–	(23)
Pax5 (BSAP)^†^	Dak-Pax5	Mo α Hu	IgG_1_	Dako	F	–	97	(24)
MHC-II	SW73.2	Rat α Sh		MRI	F	–	–	(25)
CD11b	CC126	Mo α Bv	IgG_2b_	Bio-Rad	Z	Yes (26)	–	(27)
CD163	EDHu-1	Mo α Hu	IgG_1_	Bio-Rad	F	Yes (28)	82	(29)
CD68	EBM11	Mo α Hu	IgG_1_	Dako	I	Yes (30)	–	(31)
CD169 (Siglec-1)^†^	HSn 7D2	Mo α Hu	IgG_1_	Santa Cruz Biotech	Z	–	76	(32)
Fascin^†^	55K-2	Mo α Hu	IgG_1_	Dako	F	–	93	(33)
CD83^†^	HB15e	Mo α Hu	IgG_1_	Bio-Rad	F	–	73	(34)
CD208 (DC-LAMP)	1010E1.01	Rat α Mo	IgG_2a_	Dendritics	F	Yes (35)	–	(36)
CD45	1.11.32	Mo α Sh	IgG_1_	Bio-Rad	Z	–	–	(37)
FDC^†^	CNA.42	Mo α Hu	IgM	Dako	F	Tested in cattle (38)	–	(39)
CD54 (ICAM-1) ^†^	117G12	Mo α Hu	IgG_1_	Dendritics	Z	Stated by the vendor	56	(40)
CD321 (JAM-A) ^†^		Rb α Hu	PL	Invitrogen	Z	–	75
S100^†^		Rb α Bv	PL	Dako	F	–	100
Desmin	DE-U-10	Mo α Hu	IgG_1_	Sigma-Aldrich	F	Yes (41)	–	(42)
Desmin^†^		Rb α Mo	PL	Abcam	F	–	97
Beta-actin^†^	13E5	Rb α Hu	IgG	Cell Signaling	F	–	100
Smooth muscle actin	1A4	Mo α Hu	IgG_2a_	Dako	F	Yes (43)	–	(44)
Podoplanin (gp38) ^†^	D2-40	Mo α Hu	IgG_1k_	Dako	I	–	50	(45)
Von Willebrand Factor^†^		Rb α Hu	PL	Dako	F	–	82	(46)
Vimentin	Vim3B4	Mo α Hu	IgG_2ak_	Dako	F	Yes (43)	–	(47)
Perlecan^†^	A71	Mo α Bv	IgG_1_	Source Bioscience	F	–	97	(48)
PLVAP^†^	MECA-32	Rt α Mo	IgG_2a_	Bio-Rad	F	–	74	(49)
Ki-67	MIB-1	Mo α Hu	IgG_1_	Dako	F	(50)	–	(51)

† *Antibody newly tested in sheep in this study*.

a *Mo, mouse; Hu, human; Rb, rabbit; Sh, sheep; Bv, cattle; Gt, goat*.

b *PL, polyclonal*.

c *MRI, Moredun Research Institute–Penicuik*.

d *F, signal in formalin-fixed tissues; Z, signal in zinc salts fixed tissue; I, inconsistent results*.

‡ *Numbers refer to reference/s describing sheep cross-reactivity*.

* *Cytoplasmic region of the CD3ε chain*.

### Immunofluorescence

Tissue sections were treated as previously described for IHC with the following modifications. After primary antibody incubation, sections were rinsed in PBS-T20, and incubated with an appropriate combination of anti-mouse, rabbit or rat species- or isotype-specific fluorescently labeled secondary antibodies (AlexaFluor 488, 555, 594, 633; Thermo Fisher) for 1 h at RT. Excess secondary antibodies was removed by PBS-T20 washes and sections were mounted using a liquid antifade mounting media (Prolong Gold, Thermo Fisher) containing 4′,6-diamidino-2-phenylindole (DAPI) for chromatin staining. The sections were stored at 4°C and analyzed within a week from preparation. Fluorescently labeled samples were examined using a Zeiss LSM710 confocal microscope. Images were captured and processed using Zeiss Zen software.

### Criteria Employed for the Screening of Antibodies

We screened a panel of 54 antibodies to identify cell subpopulations present in the sheep LN fixed in either formalin or zinc salt solution. Each working antibody was tested in LNs coming from a minimum of 3 different individuals to confirm reactivity. We tested both antibodies that were described as specific or cross-reactive with sheep antigens and others which were identified as specific for human/rodent cellular markers. For antibodies where cross-reactivity with ruminant targets was not stated by the manufacturers, we carried out a BLAST (basic local alignment search tool) search of the sheep genome in order to define the level of conservation of the epitope/s (or entire proteins) between sheep and the species targeted by the antibody. The reactivity of antibodies raised against proteins sharing at least 50% identity with the sheep orthologous was then tested by immunohistochemistry. Antibodies were tested initially in zinc salts fixed tissues as antigen retrieval is not required with this type of fixation (52).

To determine the presence of a positive signal we initially used antibody dilutions between 1:20 and 1:200, with the final working concentration chosen for each individual antibody based on the results of the staining. The specificity of the staining was evaluated using histological insights, primarily confirmation that the localization of the staining in the tissue matched the expected anatomical localization of the targeted cells. However, in some cases, specific signals were obtained in unexpected cell populations. In these cases the targeted cells were further characterized by co-expression of specific markers using a combination of two or three antibodies by confocal microscopy. In general, we tended to use well-recognized markers, or previously characterized ones, in association with newly identified ones.

Testing of antibodies in formalin-fixed tissues additionally included also the evaluation of different antigen retrieval techniques for the unmasking of hidden epitopes due to protein cross-linking. The combined use of multiple primary antibodies in multicolour immunofluorescence studies was limited by their reactivity using the same antigen retrieval technique and species in which they were raised, or, in case of mouse antibodies, by the isotype class. The species, isotype and concentration of the antibodies being evaluated were matched with negative control antibodies against unrelated targets to confirm specificity of staining.

## Results

Using the *in-situ* imaging techniques described above, we identified 30 antibodies capable of recognizing cell markers in fixed sheep LNs (Table 1). These antibodies provided a clear specific signal with no or minimal unspecific background staining. Eleven of the 30 antibodies solely worked on tissues fixed in zinc salts without requirement for further antigen retrieval (Table 1).

### Lymphocyte Markers

**Figure 1:**
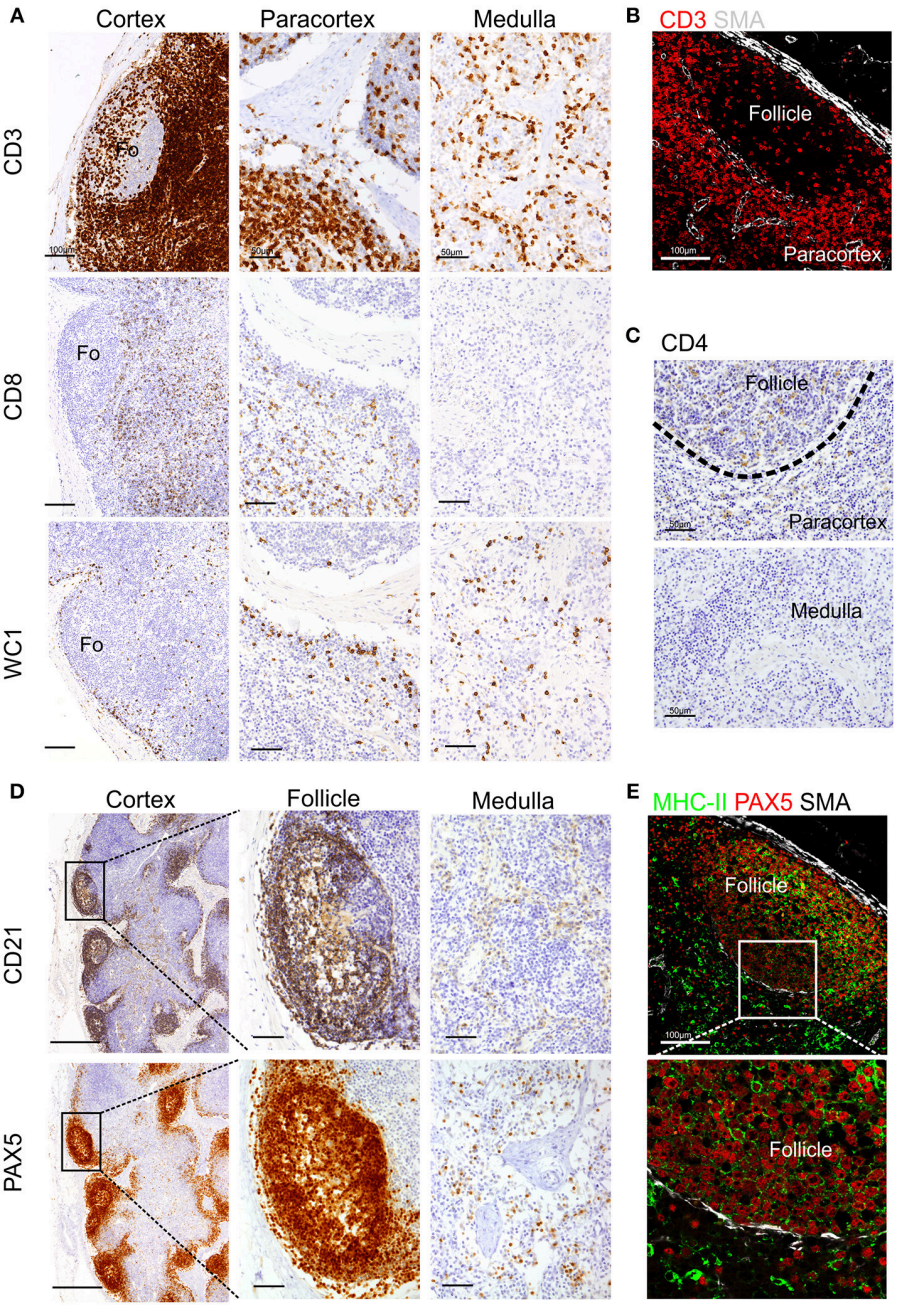
Identification of sheep lymphocytes by immunohistochemistry and immunofluorescence. Representative immunohistochemistry and confocal micrographs of sheep lymph node sections derived from paraffin-embedded tissues fixed in zinc salt solution. **(A)** Immunohistochemistry: sections were stained for CD3, CD8 (clone 38.6), or WC1 to identify T lymphocytes, cytotoxic T cells, and gamma-delta T cells, respectively. Scale bar: 100 μm for cortex and 50 μm for paracortex and medulla. **(B)** Confocal microscopy of sections stained for CD3 and smooth muscle actin (SMA, white). **(C)** Immunohistochemistry of sections stained for CD4 to identify T helper cells. Scale bar: 50 μm. **(D)** Immunohistochemistry of sequential sections stained for CD21 and PAX-5 (BSAS protein), both markers to identify sheep B lymphocytes. Scale bar: 250 μm for cortex and 50 μm for follicle inset. **(E)**. Confocal microscopy of sections stained for PAX-5 (red) and MHCII (green) expressed by B lymphocytes, a rim of SMA (white) delimits the follicle. Scale bar: 100 μm.

To detect T lymphocytes we used mAb F7.2.38 (Table 1) targeting the intracytoplasmic portion of the ε-chain of human CD3, which is highly conserved in mammals (18), and presented 92% identity with the sheep orthologue protein. This mouse mAb has been successfully used to identify CD3^+^ T cells in fixed tissue of other ruminant species closely related to sheep (16, 17). In ovine tissues, mAb F7.2.38 identifies cells that occupy the paracortical area of the LN (Figures 1A,B). As expected, some positive cells were also present along the medullary cords but not inside the medullary sinuses, nor in the subcapsular sinus or inside the trabecular sinuses. We observed lymphocyte subpopulations in similar localizations as previously described (52). For the identification of CD8^+^ T cells, we obtained overlapping results by using in zinc fixed tissue an anti-bovine mAb (CC63) cross-reactive with sheep and an anti-sheep mAb (clone 38.65 – Figure 1A): CD8^+^ T lymphocytes were evenly distributed in the cortical area but absent from the medulla as previously shown (52). We stained WC1^+^ T lymphocytes by using a sheep cross-reactive mAb (CC15), which identified γδ T cells localized in the interfollicular area and along the cortical and medullary sinuses (Figure 1A). For CD4 T cells, we used an anti-sheep mAb (44.38) that was previously reported to work in zinc salt fixed tissues (53), allowing the identification of CD4^+^ lymphocytes that were mainly confined to the follicles, where they provide help to B cells, and in lower number scattered in the paracortex (Figure 1C).

We also tested antibodies directed against three different markers commonly used for the identification of B lymphocytes: CD79a, CD21, and Pax5 (Figures 1C,D). The anti-human CD79a (clone HM57) gave inconsistent staining and therefore was not considered a reliable detector for B lymphocytes in fixed and paraffin-embedded sheep LNs (data not shown). We then used a validated cross-reactive anti-bovine mAb CC21to stain the ovine CD21 (complement receptor 2, CR2) (22, 23, 54). CD21 is expressed on the membrane of both mature B cells and FDC in sheep lymphoid tissues (52, 55–57) and, in agreement with previous description, it was mainly present in the lymphoid follicles (Figure 1C). The mAb Dak-pax5 (24) targets the Pax5 protein (B cell specific activator protein; BSAP), which is expressed in the nucleus of B cells in all the maturation stages, but not in plasma cells (58). Pax5 plays an essential role in B cell commitment as it is involved in the repression of non-B lymphoid genes and the activation of genes needed for B cell differentiation (24, 58). Similarly to CD21, anti-Pax5 antibody identified B cells in the follicles of the LN but not FDC, hence we consider it a more specific marker for B cells by IHC and IF (Figure 1C). B cells express MHC Class II (MHC II) on their surface and we could confirm co-expression with Pax5 in the follicular area (Figure 1D). Potential antibodies against other pan-B cell markers used to identify B cells in other species such as CD19 for human or CD45R/B220 in mouse are not currently available for ruminant species.

### Markers for Mononuclear Phagocytes

A broad variety of markers have been used to characterize different populations of antigen presenting cells in the mouse and humans (59, 60) but there are limited data regarding subpopulations of mononuclear phagocytes in sheep LN. Hence, we tested systematically a panel of antibodies to identify dendritic cells and macrophages in sheep LNs.

**Figure 2:**
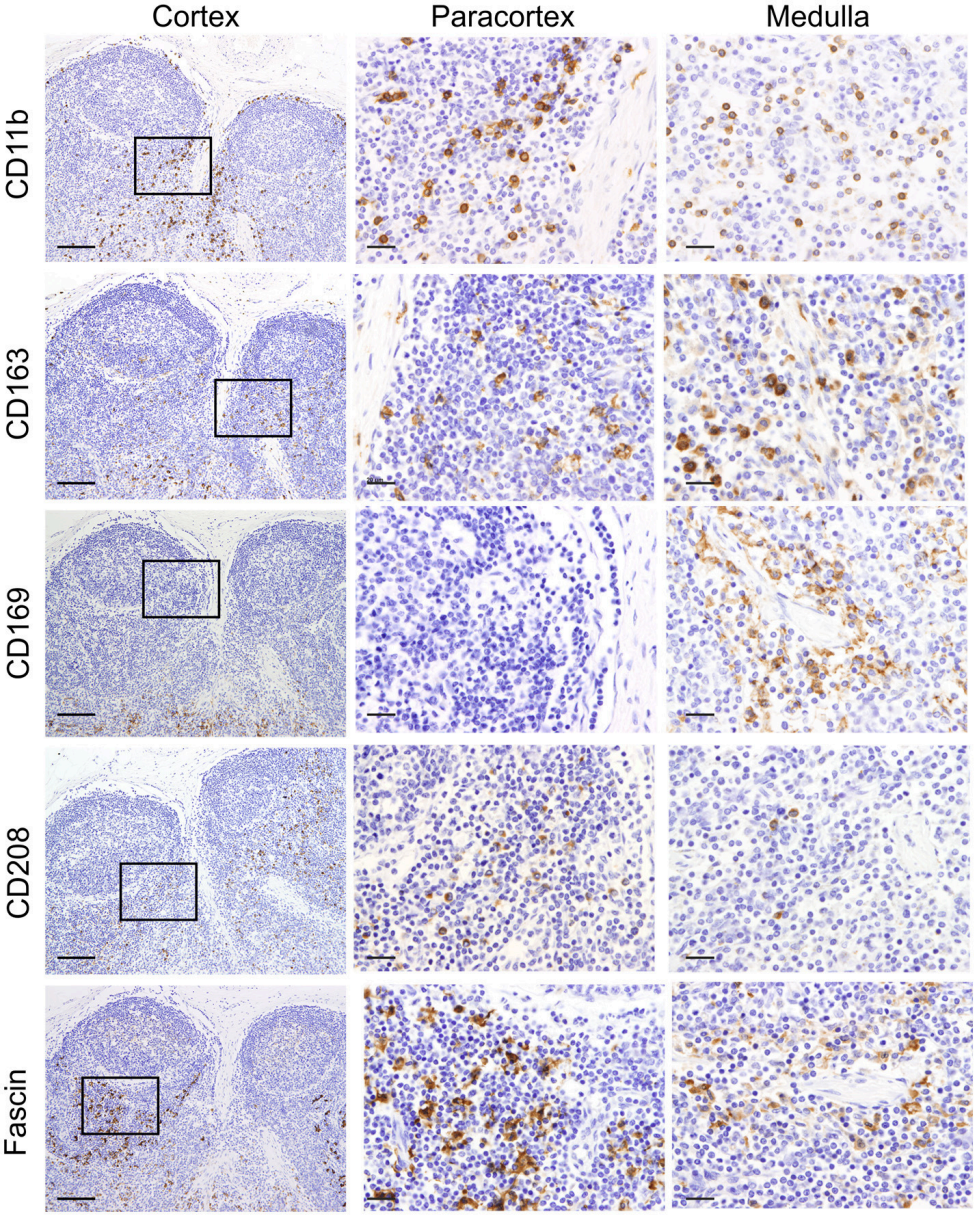
Identification of markers for sheep macrophages and dendritic cells. Representative immunohistochemistry micrographs of sheep lymph node sections fixed using zinc salt solution. Sequential tissue sections stained for CD11b, CD163, CD169, CD208, and fascin. Insets show the paracortical area at higher magnification. The expression of these markers in different anatomical areas of the lymph node allowed the identification of various populations of mononuclear phagocytes. Scale bar: 100 μm for cortex and 20 μm for paracortex and medulla.

We firstly analyzed markers putatively specific for macrophages. Different subpopulations of macrophages are generally present in the LN, where they are mainly localized along the trabecular and medullary sinuses to capture and present antigen flowing through the lymphatic system (60). CD11b (also known as MAC-1 or CR3) has been frequently associated with macrophages in sheep (61, 62). As expected, CD11b was expressed by a few cells localized along the trabecular and medullary sinuses but was not detected in the T cell area of the cortex (Figure 2). This observation is in agreement with previous studies reporting small numbers of cells expressing CD11b in the LN (26). CD163 is a glycoprotein belonging to the scavenger receptor cysteine-rich superfamily that in human is mostly expressed on tissue-resident macrophages (63) and in sheep is considered to be exclusively expressed by monocytes and macrophages and not expressed by neutrophils (unlike CD68 for example) (28, 64). To identify CD163 expression, we used the anti-human CD163 mAb (clone EDHu-1) previously validated in ovine tissues (28). CD163 was expressed by a few cells in the paracortical area and by numerous cells present along the trabecular and medullary sinuses (Figure 2), in a similar localisation as CD11b^+^ macrophages. The sialoadhesin CD169 (Siglec-1) is a lectin with Ig superfamily domains binding sialic acid and in the mouse lymph node is expressed by macrophages. In mouse LNs, CD169 characterizes two different types of macrophages: SCS and medullary (65). These macrophages are strategically placed along the sinuses where they are involved in antigen acquisition and their delivery to lymphocytes. Here we used the anti-human CD169 antibody clone HSn 72D, that in zinc salt fixed tissues labeled a population of macrophages present along trabecular and medullary sinuses but not in the SCS. This pattern of expression starkly contrast with what has been described in the mouse (Figure 2) (66).

We then screened antibodies targeting markers normally expressed by dendritic cells. Dendritic cells can be found in the paracortical area of the LN, where they interact with T cells for antigen presentation and activation. In humans, CD208 (Dendritic cell-lysosomal associated membrane protein, DC-LAMP) is specifically expressed by DCs upon activation (67). The anti-human CD208 antibody (clone 1010E1.01) is cross-reactive with sheep (35) and has been previously used to identify bovine dendritic cells in formalin-fixed tissues (38). In sheep LN, CD208 appeared to be expressed by two distinct cell populations residing in the paracortical area: the first population expressed CD208 abundantly in the cytoplasm, while the second population showed CD208 clustered in a limited perinuclear area (Figure 2); this latter pattern of expression was also detected in cells, likely macrophages, present along trabecular, and medullary sinuses.

Fascin has been demonstrated to be critical for antigen presentation by mature mouse and human DCs (68, 69). Indeed, fascin is considered a specific marker for mature DCs and not macrophages (70). Given the close relatedness of the human and sheep protein (93% of AA identity, Table 1), we tested the anti-human fascin mAb (clone 55-K2) in sheep LNs. As expected, the anti-fascin antibody showed a strong labeling of cells present in the paracortical area, where DC reside, highlighting the membrane processes extending outward the cell body (Figure 2). In the paracortical and medullary sinuses, fascin was also detectable in macrophages at very low levels (Figure 2).

**Figure 3:**
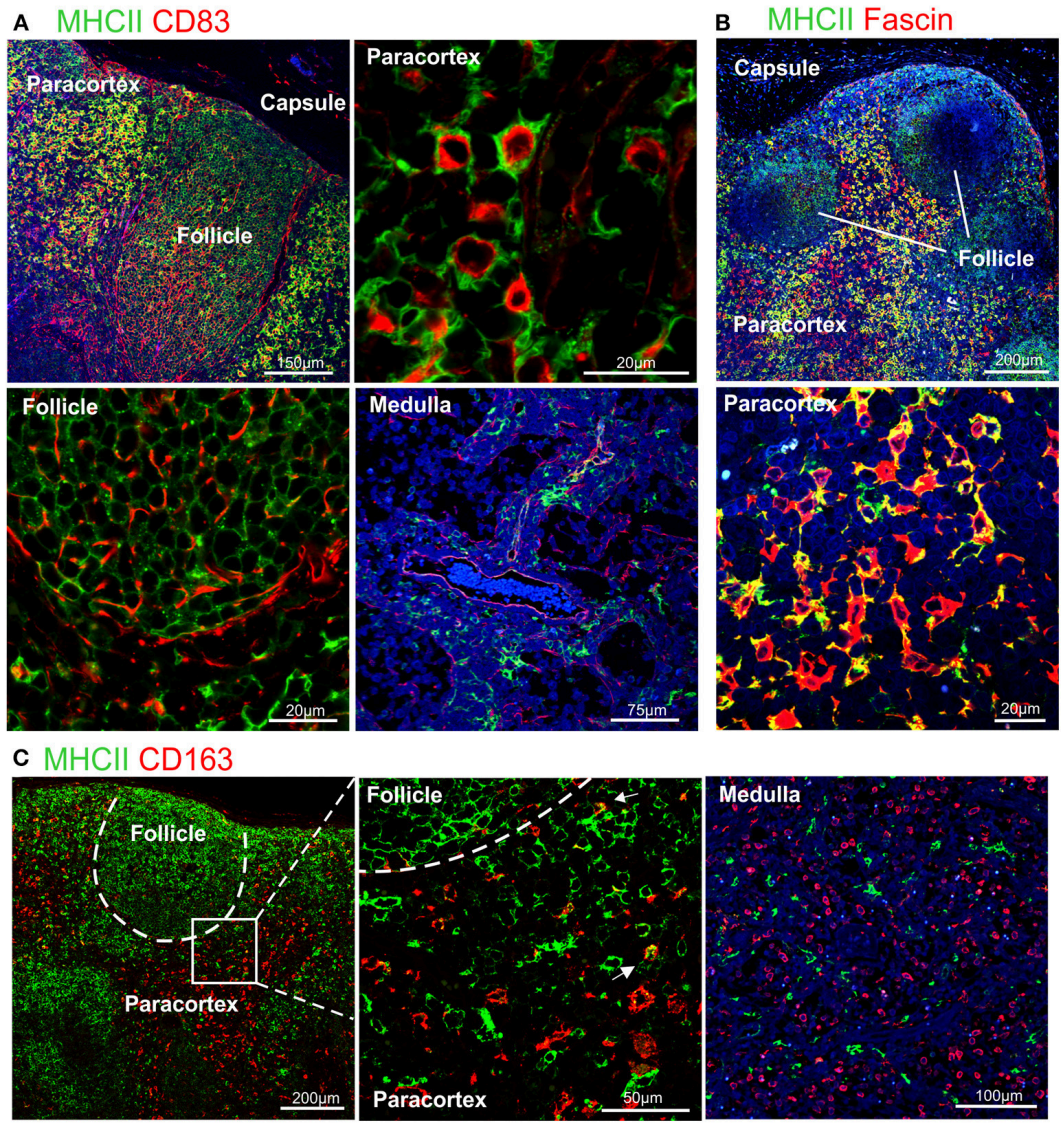
Differential expression of MHC II in dendritic cells and macrophages. Representative confocal micrographs of sheep lymph node sections. **(A)** Sections stained for MHC II (green) and CD83 (red). **(B)** Sections stained for MHC II (green) and fascin (red) to visualize the extension of DCs in the paracortical area. **(C)** Sections stained for MHC II (green) and CD163 (red). Markers expression in different areas of the lymph node is shown at higher magnification, DAPI (blue).

CD83 is broadly used as a maturation marker for human and mouse DCs (71), nevertheless it is also expressed on a variety of different cells, including monocytes, and macrophages (72), activated B and T lymphocytes (73–75) and some epithelial cell populations. CD83 expression in sheep has been described in pseudo-afferent lymph DCs (76). To test its expression in LNs, we used the anti-human CD83 mAb (HB15e). HB15e identified dendritic cells present in the paracortical area displaying a marked cytoplasmic localization, while it was not detected in macrophages located along the sinuses. In addition, the anti-human CD83 antibody labeled cells present in the B cell follicles forming a fine reticulum (Figure 3A), a staining pattern consistent with that expected for FDC.

**Figure 4:**
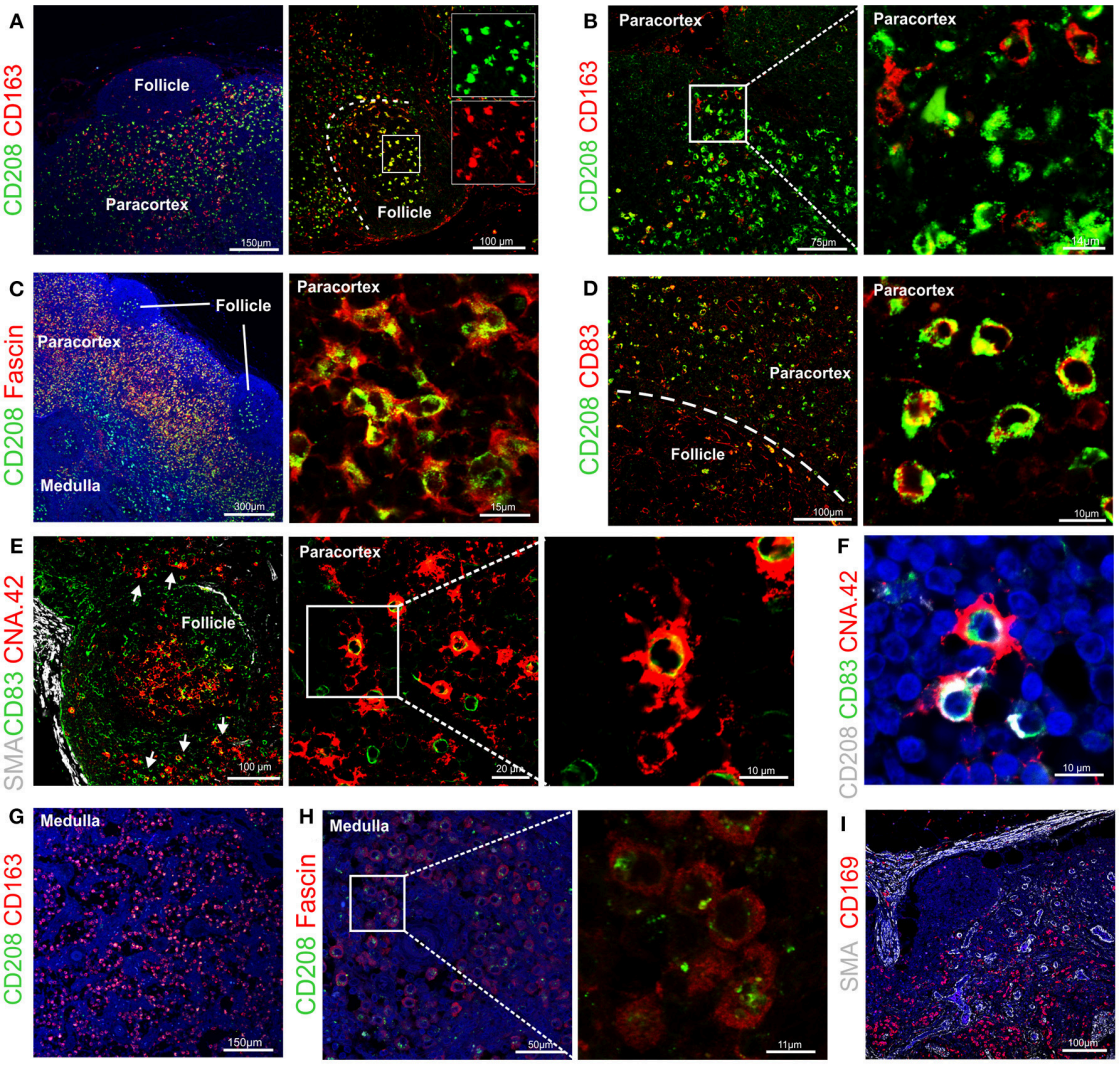
Identification of markers for sheep antigen presenting cells. Representative confocal images of sheep lymph node sections. **(A)** Sections stained for CD163 (red) and CD208 (green) identify different populations of sheep phagocytes. In the follicle, colocalization (yellow) identifies tingible body macrophages. Inset: single color at higher magnification. **(B)** Sections stained for CD163 (red) and CD208 (green) in the paracortical area of the lymph node (LN) identify different cell-subsets. **(C)** Sections stained for fascin (red) and CD208 (green), both expressed at high levels in cells residing in the paracortical area. **(D)** Sections stained for CD208 (green) and CD83 (red). Both markers are expressed in dendritic cells residing in the paracortex. **(E)** Sections stained for CD83 (green) and CNA.42 (red) showing a population of CD83^+^CAN.42^+^ dendritic cells in the paracortical area, smooth muscle actin (SMA, white) highlights the capsule of the LN. Inset: higher magnification. **(F)** Section stained for CD208 (white), CD83 (green), CNA.42 (red) to identify CD208^+^CD83^+^CNA.42^+^ dendritic cells. **(G)** Sections stained for CD163 (red) and CD208 (green), CD163 is mainly expressed in macrophages along the medullary sinuses. **(H)** Sections stained for fascin (red) and CD208 (green), macrophages in the medullary sinuses express low levels of the two markers. **(I)** Sections stained for smooth muscle actin (SMA, white), JAM-A (green) and CD169 (red). CD169^+^ macrophages are present along the trabecules but not in the subcapsular area.

To further characterize the phenotype of sheep DCs and macrophages, we combined relevant markers using multicolour confocal microscopy (Figures 3, 4). In particular, we combined newly tested antibodies with a previously validated anti-sheep MHC II to further confirm their co-expression in antigen presenting cells (Figure 3). The mAb SW73.2 (25) recognizes a monomorphic epitope on both DQ and DR beta chain of sheep and cattle MHC II constitutively expressed on antigen presenting cells such as dendritic cells, B lymphocytes, monocytes, and macrophages. In fixed LN, MHC II was expressed at high levels by interdigitating cells present in the paracortex (Figure 3) and at a lower level by the B lymphocytes present in the follicles (Figure 1E). In the medullary sinuses, macrophages expressed MHC II at lower levels (Figure 3).

CD83 was abundantly co-expressed with MHC II by cells in the paracortical area that were morphologically consistent with DCs and characterized by branched projections (Figure 3A), while the same markers were expressed at low level in the medullary macrophages. In the cortex, follicular B cells did not express CD83, in contrast to what described in human and mouse (74, 77).

High levels of fascin were also expressed in the dendrites of MHC II^high^ cells of the paracortical area (Figure 3B). These MHC II^high^ cells were negative for CD163 (Figure 3C). The CD163^+^ cells present in the cortex presented low levels of MHC II and in the medulla they appeared to be negative for MHC II (Figure 3C).

Double staining for CD163 and CD208 revealed that high expression of CD163 (a macrophage marker) and CD208 (a DC marker) appeared to be mutually exclusive in sheep LN (Figure 4A), with the exception of the follicles where both markers seems to label tingible body macrophages (Figure 4A).

By using CD163 and CD208 in combination in the paracortical area of the LN, we could identify three main different antigen presenting cell populations: CD208^+^CD163^−^, CD163^+^CD208^−^, and CD163^+^CD208^low^ (Figure 4B), which may represent different activation stages of the same cell type or distinct cell populations possessing different functions. CD208^+^ cells also stained strongly positively for fascin (Figure 4C) and for CD83 (Figure 4D) conveying a phenotype suggestive of DC. In some cases, we observed that a small population of CD208^+^CD83^+^ cells was positively labeled by the anti-human CNA.42 mAb, as described for human DC (39), which highlighted the long dendrites belonging to these cells (Figures 4E,F).

In the medulla of the LN, the double staining for CD163 and CD208 allowed the detection of a population of cells with the CD163^+^CD208^low^ phenotype (Figure 4G). Staining of these CD208^low^ cells with the anti-fascin antibody revealed low expression levels and no presence of dendrites (Figure 4H), providing indication that these cells likely belong to the monocyte-macrophage compartment. Along the medullary sinuses, we could identify CD169^+^ cells by confocal microscopy, in a similar position as CD163^+^ macrophages (Figure 4I).

### Stromal Cells Markers

In recent years, stromal cells have been recognized as having a key role in facilitating the antigen presentation process (78, 79). Stromal cells provide the supporting network of fibers that form the LN structure and produce activation cytokines (such as IL-6, IL-7, IL-15, and BAFF) and homing signals (CXCL13, CCL19, CCL21) to drive leucocytes migration. Lymphoid stromal cells are a heterogeneous population comprising groups of highly specialized cells in different areas of the LN with defined functions and phenotype (79). These distinct phenotypic and functional characteristics of stromal cells have been described in detail only in the mouse LN but not in other animal species. Here, we aimed to characterize different populations of lymphoid stromal cells of the sheep LN, and tried to identify markers that unequivocally identify them by confocal microscopy in fixed tissues.

Currently, two major subtypes of follicular dendritic cells (FDC) have been identified in the germinal centers of mice on the bases of their localization, morphology, phenotype, and function. FDCs localized in the light zone display abundant cytoplasmic extensions with a high level of membrane-bound immune complexes, whereas in the dark zone they display fewer cytoplasmic extensions and present a low capacity to trap immune complexes (80).

**Figure 5:**
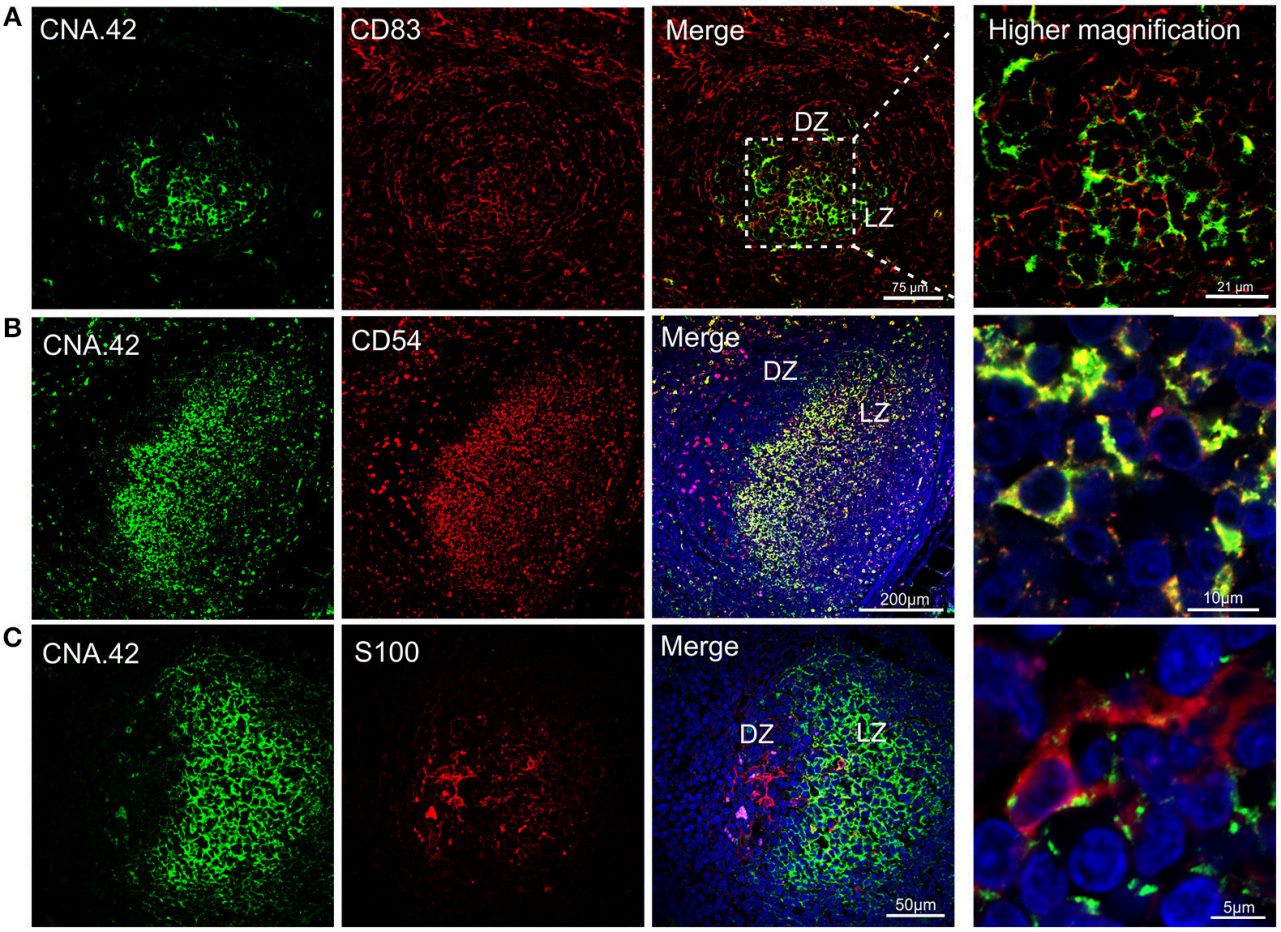
Immunophenotyping of sheep follicular dendritic cells. Representative confocal micrographs of sheep lymph node sections. **(A)** Sections were stained for CD83 (red) and CNA.42 (green). CD83 identified dark zone reticular cells present in the follicle. CNA.42 was expressed only by FDC in the light zone of the follicle. The use of CD83 and CNA.42 allow identifying a dark (DZ) and a light zone (LZ) in the follicles. Note the expression of both markers in cells present at the border between dark and light zone. **(B)** Sections stained for CD54 (red) and CNA.42 (green). CD54 and CNA.42 are both markers for light zone FDC. **(C)** Sections stained for S100 (red) and CNA.42 (green), S100 labels FDC in the dark zone. Nuclei are stained in blue (DAPI).

We found sheep FDCs to react positively with the anti-human CNA.42 antibody (15) (Figure 5) as already described for cattle (38). The epitope recognized by CNA.42 is a membrane-associated 120 kD antigen resistant to fixation that is expressed on FDCs. CNA.42 is commonly used in human diagnostic settings for the histological classification of malignancies of FDCs origin (such as follicular dendritic reticulum cell sarcoma and EBV-positive inflammatory pseudotumors) (39, 81, 82). In the sheep LNs under study, this antigen was also expressed by some mononuclear cells present in the paracortical area of the LN as described for human LNs (39). We identified sheep FDC as cells forming a reticular pattern in the light zone of the germinal centers. Furthermore, FDC in the light zone were positively labeled with an anti-human antibody targeting CD54 reported to work in sheep (Figure 5A). CD54 (also known as intercellular adhesion molecule 1–ICAM-1) is a transmembrane protein expressed by the endothelial cells and leukocytes which is implicated in the process of transmigration of leucocytes across lymphatic vessels (83). Both mouse and human FDCs have been already shown to express CD54 (84, 85). CNA.42 and CD54 were expressed in the same cell population in the follicles of the sheep LNs (Figure 5A).

We also detected a second population of FDC localized in the dark zone of the sheep germinal centers. These cells, have been previously described in the literature as “dark zone FDC” (dzFDC) or “dark zone reticular cells” but their function has yet to be defined (80, 86, 87). DzFDC lacked expression of the classical FDC marker CNA.42, but stained positively for CD83 and S100 (Figure 5). CD83 was also expressed on FDC in the light zone but at lower levels. Markers for FDC (such as CNA.42) and dark zone reticular cells (S100 and CD83) were co-expressed in the transition area between the two regions (Figure 5) suggesting that the dzFDC are a less specialized subpopulation of reticular cells that might share a similar origin with the light zone FDC.

**Figure 6:**
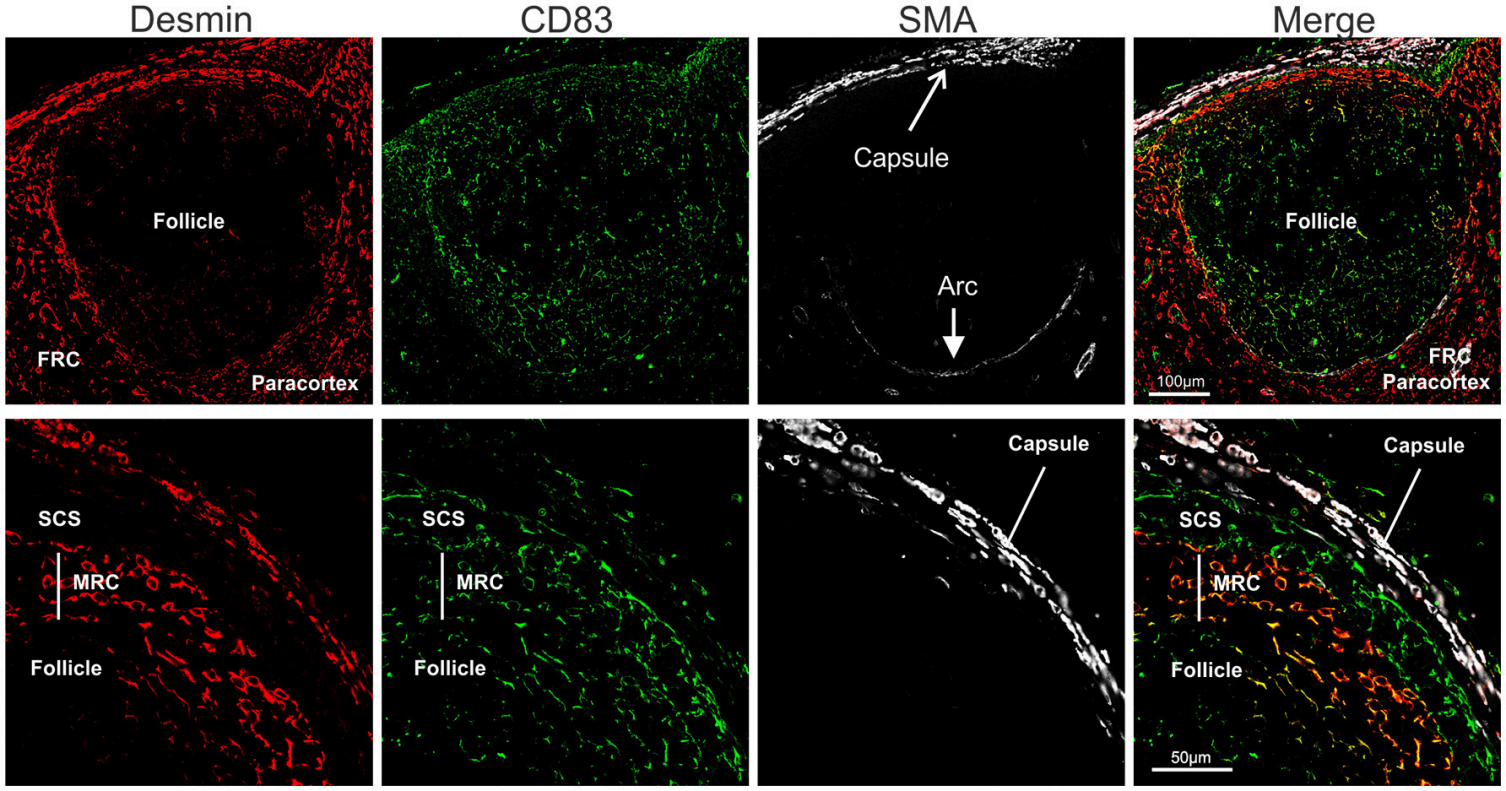
Identification of sheep fibroblastic reticular cells (FRC) and marginal reticular cells (MRC). Representative confocal micrographs of sheep lymph node sections. Sections were stained for desmin (rabbit polyclonal, red), CD83 (green), and SMA (white) to identify desmin^+^CD83^−^ FRC in the paracortex, desmin^−^CD83^+^ MRC between follicle and subcapsular sinus, and desmin^−^CD83^+^ dark zone FDC in the follicle. SMA identifies the capsule of the LN.

Stromal cells populating the paracortical and medullar area, but absent from the follicles are called fibroblastic reticular cells (FRC). We identified FRC in the sheep LN as desmin expressing cells, as previously reported for the mouse (88, 89) (Figure 6). In contrast to what observed in the mouse, we could not detect smooth muscle actin (SMA) in sheep FRC (88) but only in the LN capsule and in the pericytes surrounding high endothelial venules (HEV). Interestingly, SMA was also present on an arc-shaped line of cells delimiting the paracortical border of each follicle and separating it from the paracortex (Figure 6). We could not determine if these cells represent pericytes delineating a sinus surrounding each B cell follicle.

Furthermore, just underneath the subcapsular sinus, in correspondence with the follicles, we identified an additional population of stromal cells expressing both desmin and CD83 (Figure 6); these double positive cells (desmin^+^CD83^+^) branched from the floor of the subcapsular sinus, in continuity with sinus lining cells, toward the border of the follicles where they reached the FDCs. Due to the anatomical localization of these cells, and their expression of both FDC and FRC markers, we identified them as marginal reticular cells (MRC). These cells were recently described in the mouse LNs (90, 91) and appear to be implicated in antigen delivery to the B cells.

### Endothelial Cells Markers

**Figure 7:**
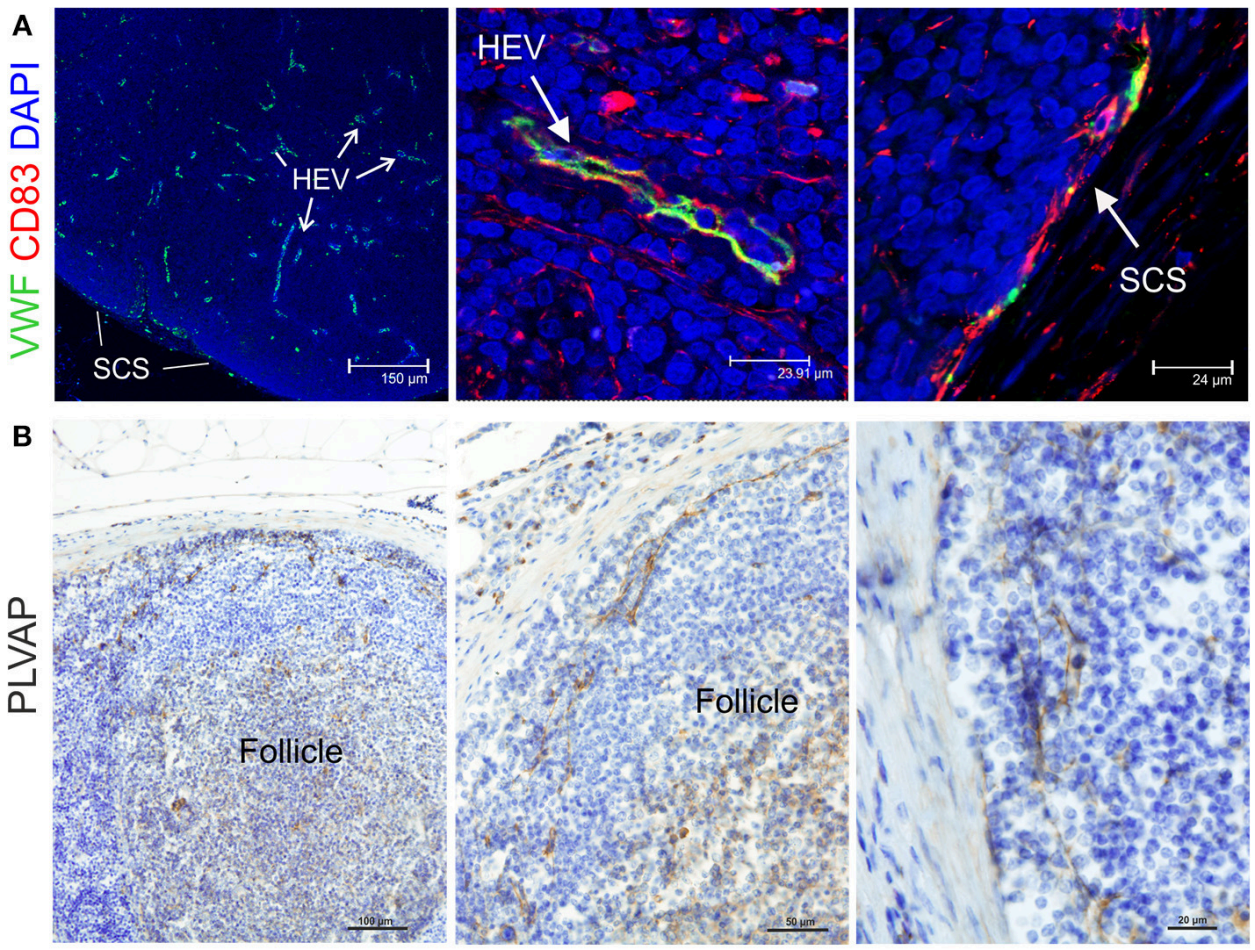
Identification of markers for sheep endothelial cells. **(A)** Representative confocal microscopy micrographs of sheep lymph node (LN) sections showing the expression of Von Willebrand Factor (VWF, green) in the cortical area of the LN. WVF expression identified cells lining high endothelial venules and subcapsular sinus. Note co-expression with CD83. **(B)** Representative immunohistochemistry micrographs of sheep LN sections collected from uninfected control animals and fixed in formalin. Sections were stained for plasmalemma vesicle associate protein (PLVAP) identifying thin cells lining the subcapsular sinus walls and branching inside the follicles underneath.

**Figure 8:**
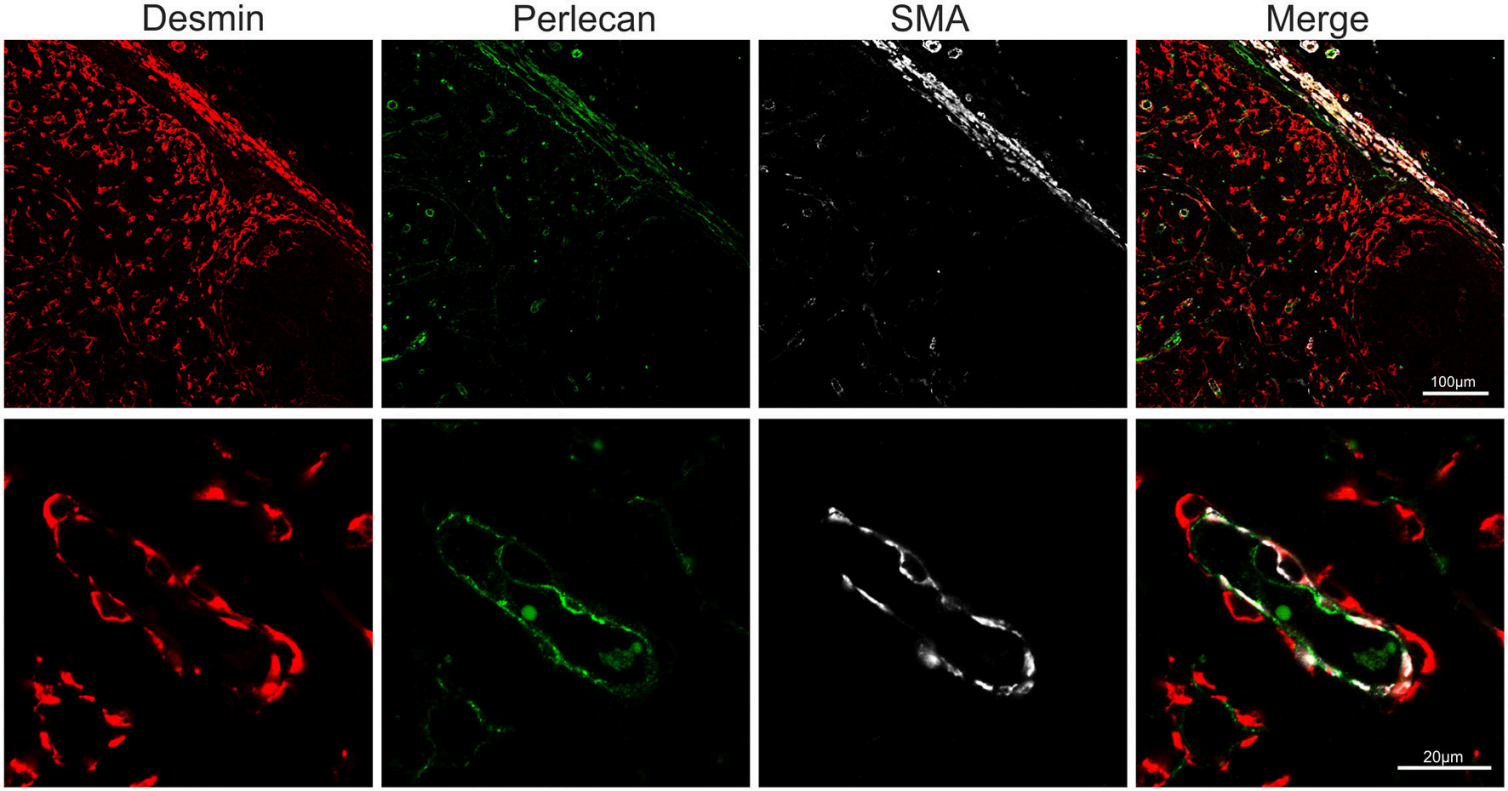
Markers for basal membrane and pericytes. Representative confocal microscopy micrograph of sheep lymph node sections stained for desmin (rabbit polyclonal, red), perlecan (green) and SMA (white) to reveal the three-layered organization of the subcapsular sinus and of the high endothelial venules in the cortical area of the lymph node.

Endothelial cells are also stromal cells and are an integral part of the LNs considering that both blood and lymphatic capillaries are critical to the anatomy and structure of this organ. We could identify blood endothelial cells (BEC) by using an anti-human Von Willebrand factor (VWF) polyclonal Ab (Figure 7A). BEC could be differentiated from lymphatic endothelial cells (LEC), which instead were localized along the SCS and expressed high levels of CD83 but low level of VWF (Figure 7A). Plasmalemma vesicle associated protein (PLVAP, a type 2 transmembrane glycoprotein that is expressed in endothelium, Figure 7B) and junction adhesion molecule-A (JAM-A, a tight-junction protein present on endothelial, and epithelial cells) were also present on the endothelial cells lining the sinus wall. In particular, PLVAP staining appeared as a delicate capillary network that from the SCS deepened in the underlying follicles, likely identifying the initial part of the conduit system (92) (Figure 7B). To further characterize the vessels in the LNs, we used an anti-bovine perlecan, also known as heparan sulfate proteoglycan 2 (HSPG2), a component of the basal membrane: this stain was able to delineate the structure of small vessels and SCS (Figure 8). The staining patterns of all of the cell populations described here are summarized in Table 2.

**Table 2:** List of cell populations identified in this study in the sheep lymph node.

**Cell type**	**Markers^**a**^**	**Localization^**b**^**	**Identified cells**
Lymphocytes	CD3	Paracortex	T cells
	CD8 CD3	Mainly in the paracortex and medullary cords	Cytotoxic T cells
	WC1 CD3	Mainly in the paracortex and medullary cords	γδ T cells
	CD4 CD3	Follicles and paracortex	T helper and T regulatory cells
	CD79^*^ Pax-5 MHC II	Follicles	B cells
	CD21 Pax-5 MHC II	Follicles	Follicular B cells
Antigen presenting cells	MHC II	Expressed at different levels in various areas of the LN	Dendritic cells, Macrophages, B cells, endothelial cells
	CD208 MHC II^high^ Fascin^high^ CD83 CD163^low/−^	Paracortex	Dendritic cells
	CNA.42 CD208 MHC II^high^ Fascin^high^ CD83	Paracortex	Dendritic cells
	CD163 MHC II^low/−^ CD208^int/−^ CD169^−^	Paracortex, few inside the SCS	Macrophages
	CD169 CD163 MHC-II Fascin^low^	Along trabecular and medullary Sinuses	Macrophages
	CD208 CD163	Follicle (2–4 per follicle)	Tingible body macrophages
Stromal cells	Desmin SMA	Capsule	Capsule
	SMA	Trabecules and around vessels	Trabecules and perycites
	Desmin	Paracortex	FRC
	Desmin CD83	Between the SCS and the follicle	MRC
	CNA.42 CD21 CD54 CD83^low/−^	Light zone of the follicle	FDC
	CD83 S100	Dark zone of the follicle	Dark zone reticular cells
	VWF^high^	Paracortex and hilum	HEV and blood vessels
	CD83 PLVAP VWF^low^	Lining subcapsular sinus, lining trabecular sinuses, enveloping sinus traversing conduits	Sinus lining cells

a *MHC II, Major Histocompatibility Complex Class II; SMA, smooth muscle actin; JAM-A, junction adhesion molecule-A; VWF, Von Willebrand factor; PLVAP, plasmalemma vesicle associated protein*.

b *FRC, Fibroblastic reticular cells; MRC, marginal reticular cells; FDC, follicular dendritic cells; HEV, high endothelial venules*.

* *Inconsistent results*.

## Discussion

Fundamental studies on immune responses in veterinary species remain hampered by a relative lack of immunological reagents. This can be rectified by the targeted production of new antibodies, molecular probes, and technologies that address specific gaps in capability for any given host species. Studies in sheep have made major contributions to our basic understanding of the ontogeny and organization of the mammalian immune system. However, in recent years investment in the development of ovine-specific immunological reagents has lagged behind most other livestock species, creating a barrier to the advancement of vaccine delivery, and disease pathogenesis studies. In this study, more than 50 antibodies, originally developed for cell markers of either ruminants or other species, were systematically tested in order to identify and localize different cell populations in fixed and paraffin embedded sheep LNs. Using the methods described in this study, we were able to successfully immunophenotype cells in the peripheral lymph node of sheep with 30 antibodies, extending the immunological toolbox for this animal species.

Importantly, we propose the existence of various populations of specialized stromal cells in the sheep lymph node, including FRC, MRC, dark zone reticular cells, and sinus lining cells. Most of these cell types have been so far described only in the mouse although they play key roles in antigen delivery and presentation (78, 79).

We identified different subsets of antigen presenting cells in the LN by using a combination of antibodies in multicolour confocal microscopy. These data complement previous studies on sheep that have mainly focused on the use of flow cytometry for the characterization of DCs collected from afferent lymph or differentiated from blood precursors (26, 61, 93–96). While the classification of ovine DC into the putative conventional DC subtypes (namely cDC1 and cDC2) and monocyte derived DC (mo-DC) focused mainly on the pool of circulating cells, relatively few studies characterized LN-resident DCs in sheep *in situ*. Recent studies have used a transcriptomic approach to close the existing gap between DC classification in different animal species (96). Unfortunately, some of the antibodies against markers that are generally used to classify these cells (such as CD11c and CD172a) did not work in fixed tissue in our hands, making it difficult to draw a direct comparison with previously identified DC subtypes. However, the differential expression of markers considered specific for antigen presenting cells still allowed the identification of potentially three different cell-subsets, residing in the cortical area (the histological location for DCs), and with morphological characteristics of DC. The first population, CD208^high^Fascin^high^MHC-II^high^CD83^+^ cells that generally displayed low levels of perinuclear CD163^−/int^, presented a phenotype compatible with classical DCs, whose function is to contribute to the activation of T lymphocytes in the paracortical area of the LN (59). The second population was less numerous and combined the expression of the classical DC markers and of CNA.42 (CNA.42^+^CD208^high^MHC-II^high^CD83^+^). Finally, a third population of CD163^+^CD208^int/−^MHC II^low^ cells was abundant in the paracortex and presented a phenotype compatible with macrophages or mo-DC due to the expression of CD163, which is considered a monocyte/macrophage marker.

Macrophages, which colonize different areas of the LN, such as the trabecular and medullary sinuses, were characterized by a lower expression of the classical DC markers (such as MHC II, fascin, and CD208) and a higher expression of CD163 or CD169. In human, CD163 is considered a marker for M2 macrophages, possessing mainly a homeostatic anti-inflammatory and tissue-repair function that distinguish them from M1 macrophages which present a pro-inflammatory phenotype (97). However, markers for macrophages polarization have not yet been defined in sheep. In some cases, CD163^+^ cells in the medullary cords do not appear to express MHC II, it is possible that very low levels of MHC II exceeded the limit of detection by confocal microscopy, as the signal of antibody binding cannot be easily amplified as with the standard HRP/AP IHC immunodetection method. Interestingly, the lack of CD169 expression along the sheep subscapular sinus, differ from what has been previously described for mouse LN. Overall, none of the antigen presenting cell markers used in this study allowed a clear identification of subcapsular macrophages in sheep LN; whether this finding reflects a lack of expression of these proteins in sheep SCS macrophages or an evolutionary difference between the two species will require further studies, possibly using RNA-probes *in situ*. Attempts to separate SCS macrophages from the tissues have been unsuccessful, and it is therefore difficult to describe the phenotype of this elusive population in detail by flow cytometry (98).

In the follicular area, apart from B cells, CD4^+^ lymphocytes, and FDC, we proposed the identification of follicular macrophages (also called tingible body macrophages), which co-expressed CD208 and CD163. CD208 is generally considered a specific marker for DC but in ruminants it has already been reported in tingible body macrophages (38).

Many more cell subtypes have been identified in the LN of the mouse and human, based on surface markers expression, cytokine profile, embryological origin, migration patterns, and antigen presentation capacity. Whenever possible, we tried to make a direct comparison with sheep subsets, but additional functional studies are required to achieve a clearer characterization of the different cell types identified in this study. In the future, the increased use of singe-cell sequencing technologies (99) and robust RNA *in situ* hybridization techniques will expand our ability to characterize rare populations of cells in the LNs of large animals and allow us to better appreciate the diversity characterizing each animal species.

## Author Contributions

EM, MC, MSR, GE, and MP: designed research; EM and MC: performed research; EM and MP: wrote the paper.

### Conflict of Interest Statement

The authors declare that the research was conducted in the absence of any commercial or financial relationships that could be construed as a potential conflict of interest.
